# Medicalising short children with growth hormone? Ethical considerations of the underlying sociocultural aspects

**DOI:** 10.1007/s11019-017-9798-6

**Published:** 2017-08-29

**Authors:** Maria Cristina Murano

**Affiliations:** Department of Culture and Communication, Linköping University, 581 83 Linköping, Sweden

**Keywords:** Bioethics, Children, Endocrinology, Growth hormone treatment, Medicalisation, Medical sociology, Philosophy of medicine

## Abstract

In 2003, the Food and Drug Administration approved the use of growth hormone treatment for idiopathic short stature children, i.e. children shorter than average due to an unknown medical cause. Given the absence of any pathological conditions, this decision has been contested as a case of medicalisation. The aim of this paper is to broaden the debate over the reasons for and against the treatment, to include considerations of the sociocultural phenomenon of the medicalisation of short stature, by means of a critical understanding of the concept of medicalisation. After defining my understanding of medicalisation and describing both the treatment and the condition of idiopathic short stature, I will problematise two fundamental issues: the medical/non-medical distinction and the debate about the goals of medicine. I will analyse them, combining perspectives of bioethics, medical sociology, philosophy of medicine and medical literature, and I will suggest that there are different levels of normativity of medicalisation. Ultimately, this study shows that: (1) the definition of idiopathic short stature, focusing only on actual height measurement, does not provide enough information to assess the need for treatment or not; (2) the analysis of the goals of medicine should be broadened to include justifications for the treatment; (3) the use of growth hormone for idiopathic short stature involves strong interests from different stakeholders. While the treatment might be beneficial for some children, it is necessary to be vigilant about possible misconduct at different levels of medicalisation.

## Introduction

In 2003, the Food and Drug Administration (FDA) approved the use of growth hormone (hGH) treatment for Idiopathic Short Stature (ISS) children, namely children shorter than average due to an unknown medical cause. Although there is no exact data on the number of children who receive the treatment nowadays in the US, it seems that, even before 2003, around one in three of all children treated were idiopathic (Voss 2006). In 2007, the European Medicines Agency (EMA) discussed and refused treatment for ISS children (EMA 2007). While there is approval for the use of hGH for recognised medical conditions, ISS has been a matter of debate (Allen and Fost 2004; Voss and Sandberg 2004; Gill 2006; Voss 2006; Rosenbloom 2010) and there is still disagreement among paediatric endocrinologists in the US on whether and when they should be treated (Frindik et al. 2010). The main concerns are: the high costs of treatment, the uncertainties about long-term side effects, and the limited (and highly subjective) height gain in adulthood. One of the main reasons to intervene is the belief that short stature might cause social and psychological burdens (Verweij and Kortmann 1997; Voss and Sandberg 2004) but opponents hold that being short is neither a medical condition nor a disease and, thus, the therapy represents cosmetic endocrinology (Voss and Sandberg 2004; Sandel 2007; Rosenbloom 2010).

Against this background, this study aims at broadening the ethical debate over the reasons for and against hGH treatment, to include considerations of the sociocultural *phenomenon* of the medicalisation of short stature, based on a critical understanding of the *concept* of medicalisation. To do so, it draws upon perspectives of bioethics, medical sociology, philosophy of medicine and medical literature. Let me now better define what I mean by medicalisation.

## Medicalisation as a phenomenon and as a concept

Medicalisation has been an object of research since the 1970s. Initially, the literature focused on the consequences of the increasing relevance that medicine has in society, both as a practice and as an institution, and it was regarded as an alarming phenomenon. For example, Irving Zola (1972) was concerned that the increasing possibility of intervening with medicine in lifestyle or conditions that previously were ‘taboo areas’ (e.g. drug addiction and pregnancy) would significantly increase individual responsibility towards health conditions (Zola 1972). Ivan Illich (1974), instead, believed that medical practice would transform human problems into technical ones and, thus, would make people less capable of dealing with these problems. Finally, Michel Foucault, during a lecture in 1974, defined modern medicine as a social practice and a bio-political strategy (Foucault 1988). He then illustrated the history of medicalisation of societies and populations, starting from the nineteenth century, which he considered the time of birth of social medicine (Foucault 1988).

In the 1990s, Peter Conrad (1992) made a shift in focus and analysed the “conceptual issues concerning medicalisation” (p. 209), defining it as a “process by which nonmedical problems become defined and treated as medical problems, usually in terms of illness and disorder” (Conrad 1992, p. 209). This definition is the starting point of the present analysis, which focuses on two fundamental issues that inform such a concept: the definition of what is medical or non-medical, and the discussion of whether medicine should have certain goals. By means of a critical reading of these two debates and reference to studies conducted in different disciplines, I will discuss the ethical issues of hGH treatment for ISS children within the socio-cultural phenomenon of the medicalisation of short stature.

Having introduced the conceptual level of the analysis, I will now define my general understanding of the phenomenon of medicalisation. Here, also, I start from Conrad’s (1992) account, according to which medicalisation is a complex and uneven sociocultural process. He believes that it should be seen in terms of degrees, rather than either/or situations, and that it can occur at different levels, i.e. conceptual, institutional and interactional ones. Halfmann (2011), in turn, makes an additional distinction between dimensions (discourse, practice and identities) and levels (micro, meso and macro). He even stresses that medicalisation may have opposite trends at the international, local and personal levels. Sometimes, the same condition might simultaneously be medicalised at one level and de-medicalised at another (Halfmann 2011). Thus, he believes that it is neither desirable nor useful to ask ourselves whether a condition is medicalised or de-medicalised in a given moment; we should, rather, identify the levels and dimensions of decrease and increase in medicalisation or de-medicalisation (Halfmann 2011). For this reason, Halfmann claims, some medicalising interventions are accepted and some are not. He takes the example of abortion rights supporters, who might approve of both some medicalisation measures (e.g. the number of hospitals providing abortions) and some de-medicalisation ones (e.g. providers no longer ask for reasons for early abortion; Halfmann 2011).

Therefore, even though medicalisation is a phenomenon mostly studied in sociology, it is often a matter of ethical debate. So far, studies in bioethics have mainly focused on disproving the traditional assumption that medicalisation is bad (Purdy 2001; Sadler et al. 2009; Parens 2013), either stressing the importance of medicine in conditions such as pregnancy and childbirth (Purdy 2001) or suggesting a case-by-case analysis (Sadler et al. 2009; Parens 2013). In short, they suggest that there are good and bad forms of medicalisation (Sadler et al. 2009; Parens 2013). Nevertheless, Sadler et al. (2009) claim that a deeper bioethical appraisal of medicalisation as a social phenomenon is missing.

In this paper, I assume that referring to hGH treatment as a case of medicalisation per se does not imply any judgment about it being ethically desirable or not. Rather, I consider the concept of medicalisation as value-neutral and descriptive as it is one way of describing the use of hGH treatment for ISS children.^1^ Thus, I suspend any *a priori* ethical judgments (either positive or negative) on the treatment and on the sociocultural process in which it takes place (and, at the same time, which it contributes to determining). In other words, I consider medicalisation as a conceptual tool of analysis that, through examination of the medical/non-medical distinction and the goals of medicine, allows me to explore, in more depth, the ethical dilemmas posed by the sociocultural phenomenon of the medicalisation of short stature. Prior to this analysis, however, more information about the treatment and the ISS condition is needed.

## Growth hormone treatment for idiopathic short children

In 1958, growth hormone treatment was introduced to treat children with severe growth hormone deficiency (GHD). The therapy was intended to compensate for their low production of growth hormone due to a dysfunction of the pituitary gland. Initially, the hormone was extracted from the glands of cadavers but in 1985, it was discovered that this process might transmit Creutzfeldt–Jakob disease, an incurable degenerative neurological disease. The same year, however, the recombinant human growth hormone (hGH) was introduced. Produced in a laboratory, it could be used as a substitute for the older technique and it also ensured a larger supply and improved safety (Wit 2002). Nowadays, the FDA allows the use of hGH treatment for children with the following conditions: Turner Syndrome, chronic renal insufficiency, Prader-Willi Syndrome, children small for gestational age and ISS (FDA 2003). Only one among these indications has not been approved by the European Medicine Agency, namely ISS (EMA 2012).

Looking at the etymology, *idios* means “one’s own” and *pathos* means “suffering”. In medicine, the term ‘idiopathic’ usually refers to a disease that arises from unknown causes. Even though there is still disagreement over how to diagnose ‘Idiopathic Short Stature’ in medical practice, some definitions have been provided. For instance, Cohen et al. (2008) define it as:

a condition in which the height of an individual is more than 2SD^2^ score (SDS) below the corresponding mean height for a given age, sex and population group without evidence of systemic, endocrine, nutritional, or chromosomal abnormalities. Specifically, children with ISS have normal birth weight and are GH^3^ sufficient. ISS describes a heterogeneous group of children consisting of many presently unidentified causes of short stature (p. 4211).

In its current use, hGH treatment should start before the onset of puberty and involves daily subcutaneous injections for a period of 4–7 years (Cohen et al. 2008). Gill (2006) claims that the estimated costs of the treatment are about € 50–75 000 for 5–7 cm gained, even though there might be significant variations according to dose, frequency and “proprietary preparation used” (Gill 2006, p. 271). The total height gain after hGH therapy is highly variable depending on the dosage and individual response, which ranges from 3.5 to 7.5 cm (Cohen et al. 2008). The treatment presents the same safety profile for both ISS and other conditions treated with hGH. Long-term side effects have not been documented but surveillance is recommended for possible risk of cancer and metabolic side effects (Cohen et al. 2008).

Besides the high costs of hGH and the uncertainties over long-term side effects, the main ethical issues arise from the rationale for giving the treatment to children with unknown aetiology. The difference between ISS and other conditions, such as GHD or Prader-Willi Syndrome, is that the therapy only brings height gain to the former, while it provides some health-related benefits to the latter (Hardin et al. 2007; Wit 2002). This is controversial for two reasons: first, ISS-treated children usually remain shorter than their peers (Rosenbloom 2010). This means that if the purpose of the treatment is to make children taller, it should be considered that they will become taller in comparison to what they would have been in adulthood without the treatment, but they will likely remain shorter than their peers. Secondly, despite the assumption among physicians that short height might generate social and psychological disadvantages (Voss and Sandberg 2004), there is both an absence of a proven relation between short stature and psychological problems (Bullinger 2011; Voss and Sandberg 2004) and a scarcity of empirical data confirming an improvement in the quality of life after hGH treatment (Theunissen et al. 2002; Allen and Fost 2004; Cohen et al. 2008).

Having briefly described hGH treatment and the condition called ISS, in the next section I will turn to the concept of medicalisation and particularly to the problematic distinction between medical and non-medical conditions.

## The ethical relevance of the medical category

The concept of medicalisation is based on the distinction between medical and non-medical problems. These categories also commonly play a significant role in ethical analyses. Opponents of medicalisation consider it “wrong” because:

construing non-medical (or life or human) problems as medical problems, construing normal human variations as pathological, commits a category mistake (Parens 2013, p. 29).

In this section, I would like to problematise the ethical relevance of this distinction, investigating what the medical category includes and why this matters (ethically) for the analysis of hGH treatment. For instance, the above quote suggests that since short stature is not a medical problem, the treatment should not be approved, otherwise it would pathologise short stature. However, there are at least three considerations that are left out in this line of reasoning: (a) the distinction between medical and non-medical conditions can be understood in different ways; (b) medicalisation refers to a broad understanding of medicine; (c) medicalisation does not necessarily mean pathologisation. Below, I will describe these considerations in more detail.

- Stating that short stature is not a medical problem does not consider that stature has been an object of medical investigation in several respects over the years. First, endocrinology is a recognised branch of medicine that studies, among other things, growth hormone and, thus, children’s development and height increase. Second, the measurement of the population mean height has been a concern of public health authorities since the nineteenth century, when the normal distribution curve was introduced (Morrison 2015). Third, the association (albeit not causal relation) between height and health has been studied: relating height and mortality (Engeland et al. 2003) or height and chronic diseases (Perelman 2014). Thus, short height has been put under the medical gaze over the years, irrespective of whether approval for hGH treatment for ISS children is given or not. From a historical point of view, short stature can be included in the medical category in the same way as many other conditions or aspects of daily life that would not commonly be defined as medical conditions, such as menopause, pregnancy or sleeping habits.
- Let us now consider the following definition:
  [medicalisation] consists of defining a problem in medical terms, using medical language to describe a problem, adopting a medical framework to understand a problem, or using a medical intervention to ‘treat’ it (Conrad 1992, p. 211).
  This quote illustrates that the concept of medicalisation implies a broad understanding of medicine, and a complex intertwinement of the medical and non-medical dimensions. According to this definition, short stature has been medicalised as follows:
  being shorter than average has been defined in medical terms as it requires medical check-ups and, at the same time, ISS is considered a statistical rather than a medical definition;
  short stature has been described using medical language by endocrinologists but measurements are made with both statistical and biochemical tools to confront the phenotype with the level of hGH present in the blood (Morrison 2015);
  a medical framework (i.e. medical check-ups) is used to understand short stature. Nonetheless, the causes of height variations are still unknown and genome-wide association studies (GWASs) have been exploring the genes that determine it (Wit 2011);
  even before hGH therapy was available, nutritionists’ and dietary guidelines were developed to maximise growth.
  Sometimes, this broad understanding of medicalisation is further expanded to include any practice that is “justified in terms of health or illness considerations” (Sadler et al. 2009, p. 414). To take an example, running daily with the aim of reducing blood pressure or to stay fit is a way of medicalising this exercise, while doing it just to enjoy a moment outdoors is not (Sadler et al. 2009).
- Medicalisation and pathologisation are not necessarily the same. With this, I make a distinction between how Conrad defines the literal meaning of medicalisation, i.e. “to make medical” (Conrad 1992, p. 210), and what pathologisation means in my interpretation, i.e. ‘to make a disease’. As Purdy highlights, medical treatments might also be offered for conditions that are not diseases, “but merely the expression of human diversity” (Purdy 2001, p. 249). For instance, when Post-Traumatic Stress Disorder was recognised in 1980, it was defined as “the normal response to an abnormal event” (Fassin 2011, p. 89). At once, it was put into the realm of psychiatry and defined as normal. In a way, short stature has also been both medicalised and depathologised. Taking hGH treatment implies regular medical examinations and the constant monitoring of growth and metabolic functioning for a period of 4–7 years. This clearly means ‘to make medical’, namely to medicalise. However, ISS was depathologised in 2007, when it was agreed to consider it a “statistical (auxologic) rather than a medical (pathologic) deviation from the norm” (Noeker 2009, p. 75). The idea that medical interventions do not necessarily pathologise the treated condition is further illustrated by other cases in which medicine is used for non-therapeutic purposes. For example, people who resort to rhinoplasty surgery for aesthetic reasons do not see their nose as a pathology, and women in menopause might take drugs and undergo regular medical check-ups, yet still define themselves as healthy.

Considering the complexity of the medical involvement with short stature revealed by these three considerations, it becomes problematic to frame the ethical analysis of hGH treatment within the dualistic approach suggested by the medical/non-medical distinction. First, the medical category is not relevant per se but it requires the assessment of short stature as a condition that *should* be treated or not; second, the involvement of medicine in the case of hGH treatment should be analysed in its own specificity, since not every medical interference with short stature is ethically problematic; finally, the mere use of hGH treatment to increase height (in case it is accepted) does not necessarily define short stature as a pathological condition. In other words, the medical/non-medical distinction implied by the concept of medicalisation might be misleading for the ethical analysis, if it is assumed to be non-problematic. Let us now consider the implications of this approach to the relevance of the medical category for the condition of ISS and for hGH treatment.

ISS is a statistical rather than pathological definition and the only parameter used to make the diagnosis is height measurement, after the exclusion of any pathology. This is a descriptive definition, which only considers quantitative information. As such, it does not have implications at the normative level (i.e. whether it is desirable or not to be—2SD). In his 1966 book, ‘The Normal and the Pathological’, Canguilhem had already made explicit the distinction between the concepts of norm and average. According to him, health is a functional ability of the whole organism, which consists in being able to adapt to the challenges posed by changing environmental circumstances. Being healthy means being able to create new norms to adapt to new conditions. Thus, being healthy means being normative. An anatomical characteristic such as height is not normal because it is frequent, but is frequent because it is normal. Canguilhem (1996) also draws a distinction between anomaly and abnormality: while the former is a deviation from the average, the latter is the inability to establish a new norm in the given environment. Being anomalous means being ‘atypical’, while being abnormal implies having a pathology. At the same time, having a pathology does not mean the absence of norms, but the expression of different norms of life, that do not allow all the individual’s needs to be satisfied (Canguilhem 1966).

ISS indicates a divergence from the average and it does not provide any information about two important aspects of the definition of health provided by Canguilhem: the context (which constantly challenges individual adaptability) and the content of the personal experience. Canguilhem believes that in order to define someone as healthy, their subjective judgment is needed. Being healthy depends on the patient’s evaluation according to the view that they have about their life (Canguilhem 1966). This highlights the limits of the definition of ISS uniquely in quantitative terms, as it stresses the lack of information about both the social environment and the experiences of short children. Defining a child as ISS leaves many questions open; for instance, does it make any difference if the child lives in the Netherlands or in Italy (not only for the difference in average height but also for cultural reasons)? What is the impact of childhood height, adult height and the speed of growth on the specific child? Does it make any difference if the child is predicted to reach 140 or 155 cm in adult height? In any case, we might question whether labelling a child as ISS is bad or good. On the one hand, it might be bad if it makes the child feel stigmatised and if this label is used to marginalise him/her. On the other hand, it might be good if it was a way to make the child part of a ‘community’, which made her/him feel free to talk about potential difficulties she/he might encounter in daily life. Nevertheless, the definition of a child as ISS cannot be the only criteria for the evaluation of the treatment because more qualitative aspects of short stature should be included. These qualitative aspects, besides psychological assessments, might include the cultural, experiential and social dimensions of the condition.

As already mentioned, it is also important to define the kind of involvement of medicine with short stature. Looking at the different practices that have put short stature under medical scrutiny, such as medical check-ups, endocrinology studies and the concern of public health authorities, it emerges that not all these practices encounter the same kind of ethical objections. So, making a bone age study with X-rays of the hand and wrist to assess the maturity of the child’s skeleton might be considered a medical procedure. As a diagnostic tool, X-rays require the short stature child to be examined by a doctor, who prescribes the test and analyses it. Yet, this practice does not commonly meet as many objections as hGH treatment, even though X-rays might be the first check-up that will eventually lead to the decision to treat the child, if it appears that the child still has a chance to grow and hGH treatment can be effective (lengthwise). However, even if the X-ray is a technical tool used by medical professionals for diagnostic purposes, it does not make short stature medical in the same way that hGH treatment does. In the latter case, a medical intervention aims at modifying children’s height, while in the former case an imaging test is used by doctors to monitor the child’s development.

This leads us to consider not just the question, ‘Is everything done by medical professionals medicine?’ (Nordin 1999, p. 105) but also: in what ways do different medical interventions matter ethically? Nordin (1999) suggests making a careful analysis of the nature of medicine and making a distinction between the goals of medicine and the means used by medicine. This distinction is particularly relevant for the ethical analysis of medicalisation, as it highlights that not everything done by medical professionals or with biomedical means should be evaluated in the same way. For instance, plastic surgery and governmental anti-smoking campaigns might both be considered cases of medicalisation. However, in the former case, a medical treatment aims at improving a certain understanding of health of help-seeking individuals, while in the latter, a preventive intervention aims at improving public health (Nordin 1999). Regarding hGH treatment for ISS children, it seems evident that the main ethical concerns are related to both goals and means. A more detailed discussion of the goals of medicine is provided in the section below; concerns about means, instead, are chiefly related to the uncertainties of the long-term safety of hGH treatment, the high costs and the limited height gain.

To conclude this section, I argue that the main ethical concern over using hGH treatment for ISS children is not the infringement of a prohibited territory per se but, rather, the kind of involvement of medicine and the definition of idiopathic short stature. Let us now turn to the debate about the goals that medicine should pursue.

## The ethical challenge of broadened goals of medicine

The idea that medical interventions are ethically problematic if they do not aim to cure patients appears frequently in the appraisal of new medical practices. For example, as mentioned above, arguments against the use of hGH therapy for ISS children are that it is a ‘cosmetic intervention’ (Voss 2006), ‘paediatric endo-cosmetology’ (Rosenbloom 2010) or ‘cosmetic endocrinology’ (Sandel 2007). In other words, since it pursues non-medical (or non-therapeutic) goals, it should not be allowed. If we take such a restricted view of the goals of medicine, limited to a traditional understanding of ‘cure’ and ‘healing’, the treatment becomes unacceptable: the fact that hGH therapy does not aim to cure children represents an infringement of the goals of medicine. In this section, I problematise this view and consider the ethical issues of a broader account of the goals of medicine.

First of all, let us consider the following statement:

the idea of medicalisation depends upon the notion that medicine has ‘proper’ goals, which are visible to those with knowledge of the essence of medicine (Parens 2013, p. 30).

This refers to the distinction between essentialist and constructionist approaches. According to Pellegrino (1999), constructionists argue that it is necessary to engage in social dialogue, consensus formation and political negotiation to define the goals of medicine. In contrast, essentialists believe that a constant revision of both medicine and society is problematic. If this constant revision were accepted, Pellegrino argues, healthcare could be managed for non-medical purposes, such as profit or political power. We should, instead, look at medicine as grounded in its own nature, as something real and independent from societal contingency. In this perspective, medicine came into existence because of the human experience of illness and this established its essential ends: care, cure and healing. These ends define medicine and the ethics of medicine (Pellegrino 1999). This is, again, a dualistic perspective. There might also be more nuanced positions. Constructionists, say, might agree on proper goals of medicine that are not necessarily intrinsic to medicine. Autopsies for legal purposes illustrate this point clearly. It is commonly accepted that a surgical procedure will be followed on cadavers, to ascertain the cause of death, even though the purpose is not to care, cure or heal.

Secondly, the process of medicalisation is increasingly confronted with cases where medical treatments do not simply aim at traditional understandings of healing, or curing. Clarke et al. (2003) even propose a new term, biomedicalisation, to indicate the transformations due to technical and scientific innovations in the US.^4^ They claim that these developments have produced several intertwined processes that take place at both societal and individual levels: on the one hand, with political and economic ramifications (such as the growing areas of the healthcare sector under private management or administrative centralisation); on the other hand, with, for example, the development of risk assessment tools (Clarke et al. 2003). Likewise, improvements have been made in computerisation, data-banking and medical technologies in general, and information has been transformed (e.g. on the distribution and consumption of health knowledge; Clarke et al. 2003). Moreover, Clarke et al. (2003) claim that these processes have created a cultural transformation: because of the increasing opportunities to shape and modify ourselves for different purposes, individual, collective identities and the perception of the body are changing. In their view, while medicalisation aims at normalising, biomedicalisation aims at transforming and customising: the focus is bodily and lifestyle improvement but also enhanced knowledge of individual potential pathologies (Clarke et al. 2003). Biomedicalisation, thus, broadens the goals of medicine: from healing to tailoring medicine to individual and collective needs.

Against this background, I consider hGH treatment for ISS stature as a case of biomedicalisation, which aims at customising stature. The ethical analysis therefore becomes more complex: the question is not whether it is acceptable to use medicine for non-therapeutic interventions but, rather, under what circumstances it is acceptable to customise short stature by medical means. In other words, considering again the distinction drawn by Nordin (1999) between medical goals and means, the debate is not about the goals of medicine in general but about the desirability of some medical means. Purdy (2001), in her criticism of traditional views on the goals of medicine, proposes one way to face this challenge:

decision-making about ends, enlightened by practitioners’ practical knowledge and, in some cases constrained by overriding social needs, ought generally to be firmly in patient’s hands (Purdy 2001, p. 258).

Purdy, thus, suggests we should “use medicalisation for genuine empowerment” (Purdy 2001, p. 261). The idea of empowerment makes even clearer the shift in focus from the goals of medicine to individual preferences. Arguments in favour of the use of hGH therapy for ISS seem to go in this direction: they make it possible for families to make their choice about the possibility of modifying height. However, the treatment should be given to children before puberty and so it primarily concerns minors in a vulnerable phase of development. How to ‘empower’ these children and make them active subjects in this kind of choice? The kind of understanding that children of that age group have about the relevance of short stature in their lives and the influence that parents might have in shaping their preferences should first be investigated, and then the best ways to involve them in this decision-making process should be explored.

As mentioned above, promoters of hGH therapy mainly refer to the psychological and social advantages that height increase might bring to children, even though this assumption is not based on empirical (psychological) evidence. However, there are some sociocultural factors worth considering. For example, an Italian study suggests that in the literature about marriage and partner choice, it is commonly accepted that there is a lower chance of marriage for men who are considered short in comparison to the average height (Manfredini et al. 2013). It also argues that in economic terms, this is partly explained by the lower socioeconomic status that is associated with men of short stature (Manfredini et al. 2013). While this can be questioned, it points to some social beliefs and assumptions about short stature that might influence decisions about the treatment. Even though the average height varies significantly according to gender and population groups and the causes of such variations are still unknown, some cultural norms have also been reported about tallness. Cohen and Cosgrove (2009), for instance, claim that in the 1950s in the USA, tall girls were considered less attractive and had a lower chance of finding a partner. They also claim that this was the main reason why mothers, in particular, would consider using hormonal therapy with oestrogen to reduce tall girls’ height. Interestingly, even if there is no formal psychological assessment of the relevance of height for women, ‘psychological indications’ are the main reason for the treatment (Rayner et al. 2010; Pyett et al. 2005).

In this context, I take the debate on the goals of medicine as a point of departure for considerations of both the sociocultural factors that influence medical decisions that are not intended to cure (in the traditional sense) a disease, and the ways and means of involvement of minors in such decisions. In the case of oestrogen treatment for tall girls, as an example, it has been reported both that adult height bears little relationship to how girls feel about the decision to use oestrogen and that not having control over the decision generates discomfort (Pyett et al. 2005). Moreover, if in the case of hGH treatment common beliefs and assumptions might play a decisive role in the decision-making process about children’s treatments, it is important to examine them critically and to raise awareness at the social level of possible social disadvantage and/or discrimination that children of a height divergent from the mean might experience. An ethical analysis of the broadened goals of medicine needs to include a broader sociocultural dimension. In other words, I propose a constructionist approach to the goals of medicine, which engages in a dialogue about sociocultural values. I also take a step further. The involvement of the sociocultural dimension not only allows a better understanding of the case; it also highlights the need to discuss the justification for the treatment and, thus, reflect on what sociocultural beliefs it is based on. In the case of hGH then, the question should not be limited to what is desirable for medicine to do, but should also consider what are desirable values to foster in society. The treatment might be given for different reasons. So, parents might wish to have a taller child because of an aesthetic preference, because of the worry about potential future distress or because of the child’s current limitations in daily life. The debate over the goals of medicine could be undertaken considering these different cases and the significance that they might have at the sociocultural level. For example, Louhiala (2007) states that, often, mothers’ negative experiences with tall stature justifies oestrogen treatment for girls predicted to be *very* tall, even if the girls do not have an opinion about it. This justification does not consider the two important aspects emphasised by Canguilhem (1966): the *context* in which the girls will grow up and their personal *experiences*. Mothers’ worries are based on their own personal and situated experiences, which do not necessarily coincide with those of their daughters. In this way, they might even influence their daughters’ wishes, and somehow predetermine their experiences in a context that is necessarily unknown to the mothers. Not only might the societal importance of stature change in the time the child grows up but also, the child might choose a lifestyle or a career for which being tall might be an advantage. Therefore, I suggest that justification for the treatment should be based on considerations of the actual condition of the child and her or his wishes. The likelihood of negative impacts that short or tall stature might have for children should be assessed leaving aside speculations of unknown variables or assumptions based on others’ experiences because they risk fostering societal prejudices (if any exist) rather than giving children the tools to accept their own body. Let us further problematise these issues in the next section, by discussing the normativity of medicalisation.

## The normativity of medicalisation

Turning now to the normativity of medicalisation, the initial assumption of this paper was that the concept of medicalisation can be considered as a value-neutral description of the sociocultural process that puts short stature under the medical gaze. While the concept is descriptive, the phenomenon commonly has some ethical implications that should be considered. Following on from the critical discussion of the conceptual grounds of medicalisation in relation to the treatment, I now consider a bad and a good example of medicalising ISS children with hGH treatment. In the former case, (a) medicalising ISS is bad if ambiguities in the definition of ISS are used instrumentally to justify the treatment. In the latter case, (b) some extremely short stature children might benefit from gaining a few centimetres.

- There is, usually, no ethical objection to the treatment of children with GHD. However, in practice, determining whether a child should be considered GHD or not may be a contentious issue. The definition of GHD itself is controversial, elusive (Allen and Fost 2004) and arbitrary (Rosenbloom 2009) because the level of GH expected by stimulation tests has been established conventionally. In some cases, children with isolated partial hormonal deficiency might even be defined as ISS rather than GHD (Cohen et al. 2008). The definition of ISS is also divisive because it is a definition by exclusion and there is disagreement on the inclusion criteria. Current knowledge only allows us to make a diagnosis after having ruled out pathological conditions, such as severe GHD, dysmorphic syndrome and skeletal dysplasia, or children born small for gestational age (Wit 2011). However, while some scholars claim that Familial Short Stature (FSS) and Constitutional Delay in Growth and Puberty (CDGP) should be included in the definition of ISS (Wit et al. 2008), others exclude them (Kelnar et al. 1999; Rosenbloom 2009)^5^. One consideration against the inclusion of FSS and CDGP is that “approximately 60–80% of all short children at or below −2 SDS fit the definition of ISS” (Cohen et al. 2008). These controversies are relevant not only at the definitional level but also, and especially, for the children’s entitlement to the treatment. If a child has GHD, he/she is more likely to be treated than a child with ISS. Similarly, an ISS child would more likely get the treatment than a FSS child. In other words, definitions have a justificatory power for the treatment, and it would be a bad form of medicalisation to describe the condition of a patient with one definition rather than another with the purpose of justifying the treatment.
- Little research focuses on the possible physical limitations of children who are −4 SDS (Wheeler et al. 2004). However, it has been shown that these children might have functional impairments or physical restrictions, i.e. they might be unable to use school bathrooms or reach elevator buttons or may have difficulty in climbing stairs (Wheeler et al. 2004). If we consider that hGH treatment might cause an accelerated short-term growth rate (and not only greater final height), in such cases it seems that the treatment might bring some benefits to the children, as it might facilitate their performance of daily tasks. In these cases, the use of hGH injections might be justified. This does not suggest that hGH treatment should always be used for ISS children who are extremely short but individual cases should be evaluated to assess whether it is the best choice for the individual child.

There is another matter to consider: the different levels of normativity of medicalisation. Mordacci (1995) illustrates this point clearly, highlighting that different definitions of health express specific ways of understanding norms. For instance, Boorse considers a norm as a statistical average. His biostatistical theory of health is descriptive and, as such, it does not make any normative claim. He defines health in terms of statistical normality for an individual’s organism, according to normal species design, and he focuses on single organ and biological functioning (Boorse 1977). This is not the only possible interpretation. To take another, Mordacci (1995) defines Nordenfelt’s theory of health as an example of a norm as desirability: “in terms of an individual’s desires or wants” (p. 483). Nordenfelt (2007) defines a person (A) as completely healthy if, “and only if, A has the ability, given standard circumstances, to reach all his or her vital goals” (p. 7). This is called the ‘holistic approach’ and it requires looking at the general development of bodily and mental states (Nordenfelt 2007). Therefore, Mordacci proposes to look at different concepts of health as interpretations of an “original common experience whose meaning can be intersubjectively recognized” (Mordacci 1995, p. 476). He claims that health is, first, a matter of experience, while its definition is an interpretation of such an experience. This interpretation is relevant at the normative level, because:

Different levels of meaning thus correspond to different kinds of normativeness (biological, mental, social, political, moral), but every normative level should be held clearly distinct from each other, as a different relationship of the various forms and dimensions of experience (bodily, mental, social or moral) to the original common source of meaning (the experience of the health of the person as a whole) (Mordacci 1995, p. 490).

Against this background, we can recognise different levels of normativity in the debate about the medicalisation of ISS with hGH treatment. Let us consider the regulatory, pharmaceutical and individual levels.

First, the FDA approval to use hGH therapy for ISS establishes a norm, according to which access to the treatment is extended to ISS children. However, while the definition of ISS includes children who are shorter than 2 SDS, the FDA further restricted the requirements to get the treatment and approved it only for ISS children who are 2.25 SDS below the mean, with a growth velocity that allows a prediction that their adult height will be at least 2 SDS below the mean (Frindik et al. 2010). Thus, this norm does not require that all short children will need to be treated but it opens the possibility for some children to receive the treatment. Moreover, the definition of ISS that they use only refers to measurable information and they do not suggest any qualitative considerations to define the cases in which short stature should be treated.

Second, it should be considered that ISS is the largest paediatric population that might have access to hGH treatment (Finkelstein et al. 2002). Moreover, Gill (2006) claims:

All the studies on GH have been sponsored by pharmaceutical companies with all of the inherent problems of such arrangements. None of the GH studies has understandably included a placebo group (receiving sterile water infections for years would be ethically difficult to justify). None has included a nutritionally supplemented comparison group (p. 271).

While nowadays there is at least one study independent of pharmaceutical companies, which examines the cancer risks in relation to GH treatment (Swerdlow et al. 2017), ISS children might be at risk of disease mongering. I understand disease mongering as the active role of pharmaceutical companies in promoting the extension of the boundaries of illness to extend their market (Bell and Figert 2012). However, the risk that industries will prioritise profit over people’s health should be analysed as a different issue from the approval of the treatment, which highlights the need for further non-industry funded research on hGH therapy for ISS. Even though pharmaceutical companies have interests in implementing the treatment, we should consider that the expansion in the use of medicine over recent decades is the result of a complex interaction of “countervailing powers” (Busfield 2010). From this perspective, the increase in the expenditure on pharmaceutical preparations and the growth in the number of prescriptions are determined by the interplay of the pharmaceutical industry with doctors, the public, government and insurance companies (Busfield 2010). To conclude, there might be different actors who have an interest in such a drug and we might wonder if the risk of disease mongering is a legitimate reason to object to FDA approval. This is not an attempt to minimise the role of marketing strategies in inducing need on the lay public and in promoting their products. It is, rather, a reminder of the distinction between the ethical evaluation of the treatment and the possible misconduct of stakeholders.

Finally, let us consider the individual level and the arguments for or against the treatment. Two opposing justifications, to decline or take the treatment respectively, are exemplified by Micheal J. Sandel and John Harris. On one hand, Sandel (2007) argues against the treatment because parents should accept their children as they are. Treating them with hGH, he claims, would mean a lack of capability to love them with their own peculiarities. This argument does not consider the active role that children might play in such a decision. For instance, possible physical limitations might be a reason why severely short children wish to be treated. On the other hand, Harris (1992) holds that there is nothing wrong with modifying height, because height itself is a neutral trait. This position is connected to the idea of customisation and individual preferences. However, it does not consider that there might be some inequalities of access to the drug due to the high costs. Most importantly, it takes for granted that the child would interpret the treatment as something neutral. This might not be the case and children might think that there is something wrong with being short. In both cases, there are important issues that remain unaddressed and a combination of these two approaches would allow a more balanced evaluation.

To conclude, the debate about the medicalisation of short stature with hGH should consider the complex interplay of different actors and dimensions involved, including at the normative level. There might be very complex interactions and causal relations behind the possibility to medicalise and many moral subjects are involved. For example, development of the treatment has required the collaboration of different actors, such as academic scholars, pharmaceutical companies and different kinds of expertise, i.e. pathologists, biochemists, and endocrinologists (Morrison 2015). Since their interests and influences are necessarily intertwined, a comprehensive analysis of the medicalisation of short stature should consider the roles of different stakeholders in light of the diverse kinds of normative implications involved. For instance, the daily experience of children with conditions defined differently (i.e. GHD, ISS or FSS) might sometimes be comparable. An extremely short child with ISS might be exposed to bullying, psychological distress or physical limitations in the same way as a GHD child with the same height. The treatment might still not be the best solution for the particular child, but it is important to open the debate nonetheless.

## Conclusion

This study has sought to provide a critical reading of the sociocultural aspects that make short stature children possible candidates for hGH treatment. It suggests that while using hGH treatment might have beneficial effects for some children, it requires a careful evaluation of the involvement of different stakeholders, who might take advantage of the drug for their own interests. The ethical issues with the medicalisation of short stature thus concerns three main points: the downplayed role of the qualitative dimension of short stature, justifications for the treatment and the possible misconduct of stakeholders at different levels. I believe that, in some cases, certain means of medicine might be used ethically to customise undesired anatomical features, but in the case of hGH for ISS, it is very important to make sure that the decision is taken with the children involved and that the individual case is considered in its specificity, being careful not to give too much importance to uncritically assumed social beliefs, unrealistic parental expectations and economic or political interests of medical professionals and pharmaceutical companies.

This paper also shows that the medicalisation of short stature should not be considered as merely a consequence of the introduction of hGH treatment. It should, rather, be seen as the result of a broader sociocultural process. On the one hand, it is linked with the development of endocrinology and the use of growth charts, the construction of the normal distribution curve and the calculation of the standard deviation from the statistical mean. On the other hand, the meanings assigned to it are associated both with biomedical technology and with its cultural and social dimensions. In the same way that medicalisation is not an ‘either/or’ phenomenon, it cannot be considered either ethically good or bad. The fact that at some levels of analysis there might be risks of unethical conduct is not a sufficient reason in itself to refuse the treatment. The analysis should, rather, investigate the ethical conduct and the level of information of all actors and stakeholders involved. There is, additionally, a need for further research: on the long-term safety of the treatment without pharmaceutical involvement, on the experience of extremely short children, on the common perceptions of short stature, and on the best ways to involve children in decisions concerning the treatment.

## Footnotes

1 There are other ways of defining the use of hGH treatment for ISS children, according to the particular focus of the analysis, such as enhancement (e.g. Sandel 2007; Harris 1992) or pharmaceuticalisation (Morrison 2015).

2 SDS is an acronym for standard deviations score from the mean.

3 GH is an acronym for growth hormone.

4 Clarke et al. (2003) conducted their study in the US, which is of relevance because the hGH has been approved in the US but not in Europe. This leaves open the comparison of medicalisation trends in the US and in Europe.

5 The difference between FSS and CDGP children is that the former definition refers to children who are short in comparison to their peers but “remain within the expected target range for the family”, while children with CDGP are short during most of their childhood, have late puberty and may have a normal height range in adulthood (Wit et al. 2008).
